# Phospholipid Glutathione Peroxidase Overexpression Mitigates Cancer Cachexia by Protecting Muscle Mass and Lowering Inflammation

**DOI:** 10.1002/jcsm.70255

**Published:** 2026-03-19

**Authors:** Elizabeth Duggan, Jordan D. Fuqua, Bo Hagy, Constantin Georgescu, Benjamin F. Miller, Holly Van Remmen, Jacob L. Brown

**Affiliations:** ^1^ Aging and Metabolism Research Program Oklahoma Medical Research Foundation Oklahoma City Oklahoma USA; ^2^ Oklahoma City VA Medical Center Oklahoma City Oklahoma USA; ^3^ FSU Department of Health, Nutrition, and Food Sciences Florida State University Tallahassee Florida USA

**Keywords:** atrophy, GPx4, lipid peroxidation, lipoxygenase, oxylipin, skeletal muscle

## Abstract

**Background:**

Cancer cachexia is a muscle wasting syndrome that occurs in ~80% of cancer patients and is the primary cause of death for 22%–30% of cancer patients. The primary challenge associated with cancer cachexia is that effective therapies to treat the associated muscle loss and dysfunction are lacking. Research exploring whether reactive oxygen species (ROS, i.e., superoxide anion and hydrogen peroxide) contributes to cancer cachexia has had mixed results. Lipid peroxidation is an underexplored component of oxidative stress that may contribute to cancer cachexia as markers of lipid peroxidation such as 4‐hydroxyneoneal (4‐HNE) and MDA (Malondialdehyde) are higher in muscle from tumour‐bearing mice when compared to controls. Phospholipid hydroperoxide glutathione peroxidase (GPx4) is an antioxidant enzyme that reduces lipid hydroperoxides. We hypothesized that reducing lipid peroxidation via GPx4 overexpression would mitigate cancer cachexia in tumour‐bearing mice.

**Methods:**

One million Lewis lung carcinoma (LLC) cells or phosphate‐buffered saline was injected into the hind flank of wildtype or GPx4 transgenic (Tg) mice at 6 months of age and the tumour developed for 4 weeks. Muscle mass, contractile function, mitochondrial respiration, RNA‐sequencing, inflammation and the oxylipin profile were assessed.

**Results:**

Muscle mass and myofiber cross‐sectional area were reduced ~25% in wildtype tumour‐bearing mice compared to control mice but not changed in GPx4 Tg tumour‐bearing mice. GPx4 overexpression (~3‐fold) did not raise maximal or specific muscle force generation in LLC‐tumour‐bearing mice. Muscle mitochondrial respiration was reduced in wildtype tumour‐bearing mice by ~40% when compared to control mice but not altered in tumour‐bearing GPx4 Tg mice. Quadricep RNA seq analysis revealed that expression of inflammatory genes was elevated in wildtype tumour‐bearing mice when compared to control mice, and the expression of these genes was reduced in tumour‐bearing GPx4 Tg mice compared to wildtype tumour‐bearing mice. Next, we found that protein content of IL‐6 was ~5‐fold greater in muscle from wildtype tumour‐bearing mice compared to control mice, and GPx4 overexpression prevented this increase in IL‐6. We assessed the muscle oxylipin profile and found that many oxylipins generated by 12/15‐Lox were elevated in tumour‐bearing mice but not impacted by GPx4 overexpression.

**Conclusions:**

Our results show that GPx4 overexpression protected muscle mass and mitochondrial respiration in tumour‐bearing mice, possibly by reducing muscle inflammation. Future studies will explore the potential mechanisms for the protective effect of GPx4 in cancer cachexia.

## 1 Introduction

Cancer cachexia is a muscle wasting syndrome that occurs in ~80% of cancer patients and is the primary cause of mortality for 22%–30% of these patients [1]. Cancer cachexia alters metabolism because of tumour‐derived factors, loss of anabolic stimuli and an increase in catabolism [2]. In patients suffering from cancer cachexia, metabolism does not slow to conserve body mass, so conventional nutritional therapies are ineffective [2].

Effective therapies to treat cancer cachexia are lacking. Multimodal strategies that concurrently target exercise, nutrition and pharmacological strategies may be required to combat cancer cachexia [3]. One potential contributor to cancer cachexia is mitochondrial hydroperoxides [4]. The generation of mitochondrial hydroperoxides, and concomitant mitochondrial protein oxidation, preceded muscle atrophy in tumour‐bearing mice [4]. Elevated mitochondrial hydroperoxide generation is associated with muscle loss and dysfunction [5]. Studies of whether reactive oxygen species (ROS, i.e., superoxide anion and hydrogen peroxide) contribute to cancer cachexia have mixed results [6]. Some studies show targeting ROS reduced expression of genes associated with atrophy in muscle [7] and the maintenance of muscle mass [8, 9] in models of cancer cachexia. Other studies show that targeting ROS does not mitigate cancer cachexia [8]. When the antioxidant enzyme CuZn Superoxide Dismutase (*Sod1*KO) is deleted in Lewis lung carcinoma (LLC) tumour‐bearing mice there is greater muscle oxidative stress [6]. However, cancer cachexia was not exacerbated in *Sod1KO* mice [6]. In fact, antioxidant supplementation can accelerate tumour growth [8]. More studies are needed to conclude whether targeting ROS could be a beneficial treatment for cancer cachexia.

Lipid peroxidation is an underexplored component of oxidative stress that may act as a mediator of cancer cachexia. Markers of lipid peroxidation such as malondialdehyde and 4‐HNE are elevated in cachectic muscle when compared to controls [10, 11]. In addition, inhibition of enzymes that can generate lipid hydroperoxides, such as cyclo‐oxygenase 1/2 [12] and 12/15‐lipoxygenase [13], protects against cancer cachexia. Phospholipid hydroperoxide glutathione peroxidase (GPx4) is an antioxidant enzyme that reduces lipid hydroperoxides predominantly within membranes using glutathione [14]. GPx4 is critical for preventing lipid hydroperoxide‐mediated cell death [14] and is required for membrane stability [15]. GPx4 is important for maintaining mitochondria [16], a critical organelle in muscle that is vital for maintaining muscle mass and strength [17]. In addition, GPx4 overexpression protects against other conditions associated with muscle atrophy and muscle dysfunction such as sarcopenia [18]. Therefore, lipid hydroperoxides may be a viable target to treat cancer cachexia.

We investigated if lipid hydroperoxides are a potential key mediator of cancer cachexia. We hypothesized that mice with GPx4 overexpression (GPx4 Tg) would be protected against cancer cachexia when compared with wildtype counterparts. To test this hypothesis, we implanted LLC tumours in wildtype and GPx4 Tg mice and measured skeletal muscle outcomes related to cancer cachexia.

## 2 Methods

### 2.1 Animals and Interventions

Animal experiments were approved by the Institutional Animal Care and Use Committees at Oklahoma Medical Research Foundation and the Oklahoma City VA and were performed at the Oklahoma Medical Research Foundation. Experimental mice were group housed, kept on a 14:10‐h light:dark cycle and had access to standard rodent chow and water ad libitum. The breeding and characterization of the GPx4 Tg mice was previously described [19]. In our hands, we have characterized the GPx4 Tg mouse in models of aging and oxidative stress‐induced frailty [18, 20]. The strain of the mice was C57Bl/6J mice. A combination of male and female mice were used for all experiments. Mice were sacrificed via CO_2_ asphyxiation followed by cervical dislocation at 6–7 months of age. Tissues were collected and snap frozen in liquid nitrogen for subsequent analyses. Muscle masses for gastrocnemius, quadricep, soleus and tibialis anterior were pooled from both hindlimbs for muscle wet weight analysis. EDL muscle was not pooled from both hindlimbs for muscle wet weight analysis because one EDL was used for contractility measurements.

### 2.2 Lewis Lung Carcinoma Growth and Tumour Implantation

Lewis lung carcinoma cells were grown and implanted as previously described [9]. LLC cells (ATCC CRL‐1642) were plated in 250‐mL culture flasks in Dulbecco's Modified Eagle's Medium supplemented with 10% foetal bovine serum plus 1% penicillin and streptomycin. Once confluent, cells were trypsinized, counted and diluted in phosphate‐buffered saline (PBS) for implantation. LLC cells were implanted into the right hind flank of the mouse. Wildtype and GPx4 Tg mice were implanted with LLC cells at ~6 months of age. LLC cells were plated at passages 2–5. Because LLC cells were suspended in 100 μL of PBS for injection, control mice were injected in the hind flank with this same volume of PBS.

### 2.3 Ex Vivo Extensor Digitorum Longus Contractility

Male and female mice were used for contractility measurements. All successful contractility assessments are shown in the dataset. If the tendon slipped out of the knot holding the EDL tendon, the sample was omitted from the dataset. EDL muscle was suspended on a dual‐mode muscle lever system (300C‐LR, Aurora Scientific Inc., Aurora, Canada) and a hook in Krebs buffer. Muscles were placed at optimal length and allowed 20 min of thermoequilibration at 32°C. A supramaximal current (600–800 mA) of 0.25‐ms pulse duration was delivered through a stimulator (701 C, Aurora Scientific Inc.), while train duration for isometric contractions was 300 ms. Data were recorded and analysed using commercial software (DMC and DMA, Aurora Scientific). Cross‐sectional area of EDL muscle was determined by the ratio of fibre length to muscle length.

### 2.4 Tibialis Anterior Muscle Cross‐Sectional Area and Fibre Typing

Tibialis anterior samples from male and female mice were randomly selected from each group. Fibre‐type percentages (based on myosin heavy chain expression) and fibre‐type specific cross‐sectional area (CSA) were performed as described by Schenk et al. [21] in Tibialis Anterior (TA) Muscle. Each muscle was frozen in embedding medium (OCT). Ten micrometer sections were cut from the frozen tissue block using a rotary microtome in a cryostat (Leica CM1850 Cryostat, Germany) at −20°C and placed on glass slides. We fixed, stained and imaged the slides as described by Schenk et al. [21]. Briefly, sections were fixed for 5 min in cold acetone at −20°C. Sections were incubated in blocking solution (5% Normal Horse Serum PBST) for 30 min at RT and then incubated at 4°C overnight with a cocktail containing antibodies against myosin heavy chains (MHC) and laminin: anti‐MHC I (BA‐F8), anti‐MHC 2A (SC‐71 s) anti‐MHC 2B (BF‐F3s) (Developmental Studies Hybridoma Bank, Iowa City, IA, USA), and antilaminin (L9393 Sigma). Samples were mounted using appropriate medium (ProlLong Glass Antifade Mountant, Invitrogen, P36980) and images were obtained via Nikon Eclipse Ti2 (Nikon, Tokyo Japan) at 10X magnification. The Nikon Eclipse Ti2 was equipped with a motorized stage, so we were able to image the entire muscle. The cross‐sectional area of the muscle fibres was measured using ImageJ software for approximately 500 fibres per sample. Individuals performing the CSA analysis were blinded and fibres spanning the entire TA muscle were represented in the CSA analysis. If the individuals analysing the fibre images were not sure whether the fibre was stained green or black, they were instructed to omit the fibre from the analysis.

### 2.5 Respiration in Permeabilized Fibre Bundles

Gastrocnemius from samples were randomly selected from each group. Small strips of red gastrocnemius muscle were teased to near‐single fibres in ice cold buffer X (7.23‐mM K2EGTA, 2.77‐mM CaK2EGTA, 20‐mM imidazole, 0.5‐mM DTT, 20‐mM taurine, 5.7‐mM ATP, 14.3‐mM PCr, 6.56‐mM MgCl2–^6^H2O and 50‐mM K‐MES with a pH of 7.1). These fibre bundles were then permeabilized with saponin for 30 min. Mitochondrial oxygen consumption rate (OCR) and hydroperoxide production rate were simultaneously measured using the Oxygraph‐2 k (O2k, OROBOROS Instruments, Innsbruck, Austria) respirometer and fluorometer as previously described [6, 18, 20]. OCR was measured in permeabilized fibre bundles in buffer Z media containing 10‐μM Amplex UltraRed (Molecular Probes, Eugene, OR), 1‐U/mL horseradish peroxidase, superoxide dismutase and blebbistatin (25 μM) at 37°C. Rates of respiration were determined using the following sequential additions of substrates and inhibitors: glutamate (10 mM), malate (2 mM), adenosine diphosphate (0.125–11.81 mM), succinate (10 mM), rotenone (1 μM), antimycin A (1 μM) and N,N,N′,N′‐tetramethyl‐p‐phenylenediamine (TMPD) (0.5 mM) immediately followed by ascorbate (5 mM). Ascorbate is added to ensure TMPD is reduced, so TMPD can continue to donate electrons. Antimycin A was subtracted out of respiration measurements to account for nonmitochondrial oxygen consumption. Data for both OCR and rates of hydroperoxide generation were normalized to muscle bundle wet weights, weighed on a scale that is accurate at 0.1 mg.

### 2.6 F_2_‐Isoprostanes

F_2_‐Isoprostanes (oxidized arachidonic acid) are lipid peroxidation products that are used as biomarkers for oxidative stress. Quadricep from three male and three female samples were randomly selected from each group. Unfortunately, there was an error in our first F_2_ isoprostane experiment, which left us with limited samples when we repeated the analysis (3–5 samples per group). Levels of F_2_‐isoprostanes in 100–150 mg of quadriceps were determined by negative ion chemical ionization gas chromatography–mass spectrometry as previously described [22]. The level of F_2_‐isoprostanes in muscle tissues was expressed as nanograms of 8‐Iso‐PGF_2α_ per gramme of muscle mass.

### 2.7 Protein Carbonyls

Protein carbonylation is an irreversible oxidative modification of proteins that adds carbonyl groups to amino acid side chains. Protein carbonyls are an oxidative stress that often occurs through lipid peroxidation products. Extensor digitorum longus from three male and three female samples were randomly selected from each group. Extensor digitorum longus protein extracts were made by sonication in 20‐mM sodium phosphate buffer, pH 6.0, with 0.5‐mM MgCl_2_, and 1‐mM EDTA. Homogenates were centrifuged at 100000 ×*g* for 1 h to obtain the cytosolic fraction. Pellets obtained after centrifugation were resuspended by sonication in P3 buffer (2% SDS, 0.5% NP40, 0.5% deoxycholate at pH 6.0) and centrifuged at 100000 ×*g* for 20 min to obtain the detergent soluble fraction. Both the fractions were labelled with FTC to measure global levels of protein carbonyls in cytosol and detergent soluble fractions. Samples were loaded onto 4%–15% gels and visualized utilizing the Typhoon 9400 (Amersham, Piscataway, NJ, USA) with excitation at 532 and emission with a 526 SP emission filter. Total carbonylated proteins were analysed against the abundance of the protein with Coomassie Blue staining and quantified using Un‐Scan‐it software (Silk Scientific, Orem, Utah, USA).

### 2.8 Lipid Analysis Was Performed at the UCSD Lipidomics Core

Gastrocnemius from three male and three female samples was randomly selected from each group. Lipid analysis was performed at the UCSD Lipidomics Core as previously described [23, 24]. The UCSD Lipidomics core prepared primary and internal standards. Metabolites were extracted from gastrocnemius tissue as described [25]. UPLC‐MS/MS was used to measure the oxylipins [23]. Analytes were validated by linearity, recovery rate, matrix effect, accuracy/precision and stability [23]. Data are presented as picomoles per milligramme tissue (pmol/mg). If an oxylipin was detected in less than half of the tissue sent to the lipidomics core, we omitted the oxylipin from our analysis.

### 2.9 RNA Isolation and Transcriptomics

Quadricep muscles were collected and frozen in liquid nitrogen at time of harvest. Quadricep from three male and three female samples were randomly selected from each group. Quadricep muscle was then powdered before subsequent analyses. Quadricep muscle, 20–30 mg, was homogenized into a 1‐mL TRIzol solution. RNA was isolated as previously described [4]. Isolated RNA purity and concentration were confirmed using BioTek (Winooski, VT) Power Wave XS plate reader with Take3 microvolume plate and Gen5 software.

Sequencing was performed on an Illumina NovaSeq 6000 instrument with paired‐end 150‐bp reads. Sequence reads were trimmed to remove possible adapter sequences and nucleotides with poor quality using Trimmomatic v.0.36. The trimmed reads were mapped to the *Mus musculus* GRCm39 (mm39) reference genome available on ENSEMBL using the STAR aligner v.2.5.2b. Unique gene hit counts were calculated by using feature Counts from the Subread package v.1.5.2.

We used R packages limma‐voom and edgeR as parts of a two‐stage pipeline for performing Differential Expression (DE) analysis of RNA‐sequencing (RNA‐seq) data. In short, the edgeR package cleans and organizes the count data, the limma package performs robust statistical testing, while the voom function translates between the two packages. Read‐count normalization and differentially expressed analyses were performed using the edgeR package from Bioconductor. Expression values quantile normalized with the voom function were analysed for differential expression using the standard functions of the limma package. Moderated *t*‐test *p*‐values were adjusted for multiple testing using the false discovery rate (FDR) method. The moderated *t*‐statistic computed by the Empirical Bayes function in the R package limma (Linear Models for Microarray Data) is a statistically enhanced version of the ordinary *t*‐statistic designed to increase the power and stability of differential expression analysis with small sample sizes. The test is moderated because it uses an Empirical Bayes approach to stabilize the gene‐wise variance estimates. Instead of relying solely on the possibly unreliable variance calculated from the small sample size of a single gene, Empirical Bayes borrows information across the thousands of other genes in the dataset, effectively shrinking each gene's estimated variance toward a common, pooled prior variance. This stabilization prevents extreme variance estimates from leading to spurious significant or nonsignificant results, thereby yielding a more powerful and reliable test statistic than the ordinary *t*‐statistic. FDR (*q*‐value) < 0.05 and absolute log2 fold change above 1 were used as criteria to filter significantly differentiated genes.

Gene Set Enrichment Analysis (GSEA) was conducted using specialized Bioconductor packages, including fgsea, ReactomePA and viewPathway, to identify functionally related gene sets (e.g., GO terms, KEGG and Reactome pathways) that were significantly overrepresented among the differentially expressed genes.

Ingenuity Pathway Analysis (IPA, QIAGEN, Redwood City CA, https://www.qiagenbioinformatics.com/products/ingenuitypathway‐analysis) was used to explore significant gene networks and pathways interactively.

### 2.10 Immunoblotting

Gastrocnemius muscle was powdered before subsequent analysis. Gastrocnemius from three male and three female samples was randomly selected from each group. ~40‐mg gastrocnemius muscle was homogenized in the Qiagen TissueLyser II in RIPA Buffer (Pierce 89 901) containing 1‐μL/mL Protease Inhibitor Cocktail (Calbiochem 539 134) and 10‐μL/mL Halt Phosphatase Inhibitor (Thermo/Halt 78 420) at a frequency of 30 Hz/s for 2 min. Homogenate concentrations were determined using the Bradford assay. Samples were loaded across two gels each gel containing three wildtype sham, three wildtype LLC, three GPx4 Transgenic Sham and three GPx4 Transgenic LLC samples. 7 μL of Laemmli loading buffer was added to 30‐μg total protein dissolved in RIPA buffer. Samples were then boiled at 95°C for 5 min to denature the proteins. Samples were resolved by sodium dodecyl sulphate‐polyacrylamide gel electrophoresis, transferred to a nitrocellulose membrane and blocked in Tris‐buffered saline, pH 7.6, (TBS) containing 5% BSA weight by volume for 90 min, shaking. Ponceau S was used to ensure protein was transferred onto membranes, and membranes stained with Ponceau S were scanned for subsequent densitometry analysis for normalization. Membranes were probed overnight for antibodies specific to Stat3 (Cell Signalling 4904), Phospho Stat3 (Cell Signalling 9145), Oxphos Complex (ThermoFisher 45‐8099), iPLA_2_ (abcam ab309595), Cox2 (ThermoFisher 35‐8200), 12/15‐Lox (abcam ab244205), 5‐Lox (cell signalling 3289), biotinylated p‐NFκB (cell signalling 3033) and NFκB (cell signalling 8242). Primary and secondary antibodies were diluted in TBS containing 3% BSA and 0.1% Tween 20 and used according to manufacturer's protocol. Membranes were washed after primary and secondary antibody (Cell Signalling, Anti‐rabbit IgG, HRP‐linked Antibody #7074) incubations 4 × 5 min in TBS containing 0.1% Tween 20 and then imaged using Syngene G Box via chemiluminescence. All bands were normalized to Ponceau S stain as a loading control. To compare across membranes, samples were normalized to the average values of all control samples (wildtype sham) on the membrane. We used AlphaView software (Protein Simple, Biotechne, Minneapolis, MN) for all densitometry analyses (proteins and ponceau S).

### 2.11 Cytokine Array

We homogenized 40 mg of quadricep muscle in ice cold phosphate‐buffered saline with halt protease inhibitor; 100 μg of protein from the quadricep muscle was used for the Proteome Profiler Mouse Cytokine Array Kit Catalogue Number ARY006 (R&D Systems, Minneapolis, MN 55413). Cytokine Array was performed per the manufacturer's protocol. Membrane was developed via chemiluminescence and imaged using Syngene G box as described in the immunoblot section. Cytokines were normalized to the internal controls found on each membrane.

### 2.12 Statistical Analysis

**FIGURE 1:**
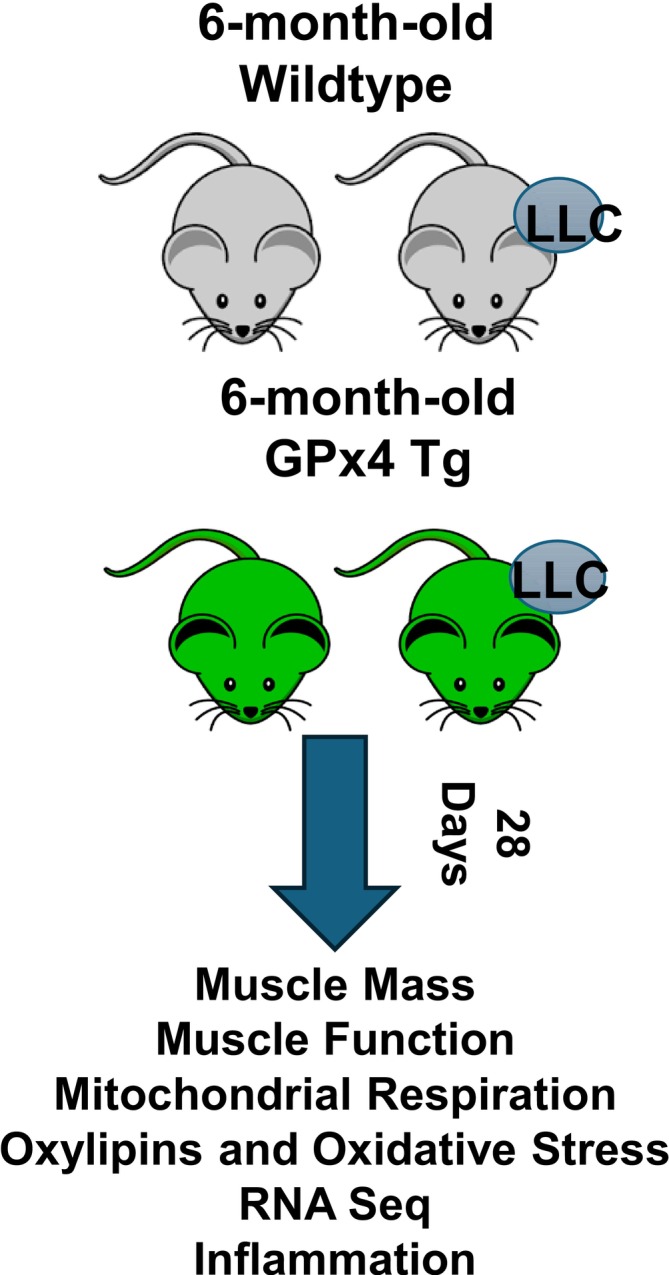
Experimental design for the study.

Figure 1 shows the study design. Data are expressed as mean ± SEM We used a two‐way ANOVA with the main effects of genotype and LLC implantation. When there were significant differences detected via an interaction, we used a Tukey–Kramer post hoc test. For all experiments, the comparison‐wise error rate, *α*, was set at 0.05 for all statistical tests. An asterisk (*) was used to denote significant differences denoted from the post hoc test. (*) indicates the p value is less than 0.05. (**) indicates the *p*‐value is less than 0.01. (***) indicates the p value is less than 0.001. All data were analysed, and graphs were compiled using GraphPad Prism (La Jolla, CA, USA).

## 3 Results

### 3.1 GPx4 Overexpression Lowers Markers of Lipid Peroxidation in LLC‐Tumour‐Bearing Mice

**FIGURE 2:**
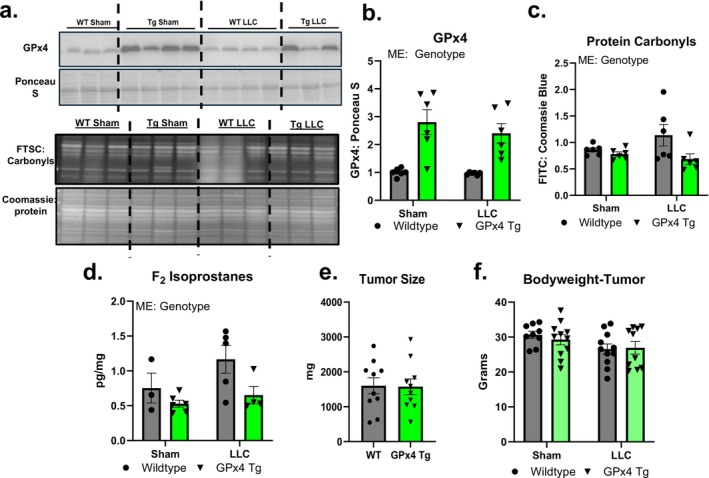
GPx4 overexpression lower lipid peroxidation. (a) Representative western blot and carbonyl images. (b) Protein content of GPx4 in gastrocnemius muscle. (c) Protein carbonyls in EDL from LLC‐tumour‐bearing wildtype and GPx4 Tg mice. (d) F_2_ isoprostanes in quadricep from LLC‐tumour‐bearing wildtype and GPx4 Tg mice. (e) Tumour size in LLC‐tumour‐bearing wildtype and GPx4 Tg mice. (f) Bodyweight with tumour wet weight subtracted in control and LLC‐tumour‐bearing wildtype and GPx4 Tg mice. An *n* = 3–11 per group was used. Asterisk denotes post hoc differences at an alpha set at *p* < 0.05. ME: main effect.

There was a main effect for GPx4 protein content in gastrocnemius muscle to be higher in the GPx4 Tg mice when compared to wildtype mice (Figure 2a,b). There was a main effect for protein carbonyls in EDL muscle and F_2_ Isoprostanes in quadricep muscle to be lower in GPx4 Tg when compared to wildtype mice (Figure 2a,c,d). Tumour size and body weight with tumour weight subtracted were not different among groups (Figure 2e,f).

**FIGURE 3:**
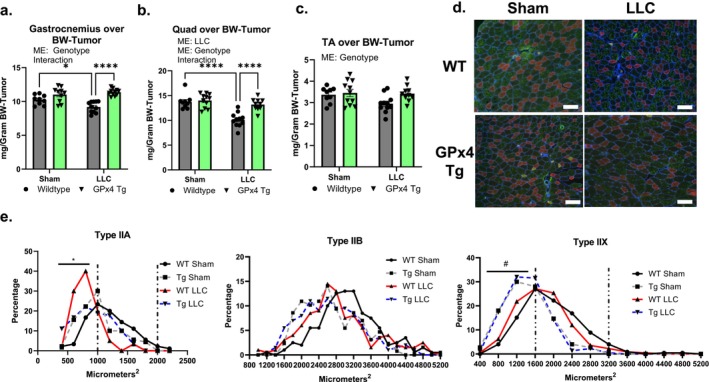
GPx4 overexpression mitigates muscle atrophy in LLC‐tumour‐bearing mice. (a) Gastrocnemius mass in control and LLC‐tumour‐bearing wildtype and GPx4 Tg mice. (b) Quadricep mass in control and LLC‐tumour‐bearing wildtype and GPx4 Tg mice. (c) TA mass in control and LLC‐tumour‐bearing wildtype and GPx4 Tg mice. (d) Representative images for CSA and fibre typing in tibialis anterior muscle. Type I: Yellow. Type IIA: Red. Type IIB: Green. Type IIx: Black/Unstained. (e) CSA distribution for different MHC isoforms in tibialis anterior muscle. Scale bar = 100 μm. An *n* = 8–11 per group was used. Asterisk denotes post hoc differences at an alpha set at *p* < 0.05. * GPx4 Tg LLC is different from Wildtype LLC. # GPx4 Tg is different when compared to wildtype. ME: main effect.

*Muscle Mass is higher in LLC‐tumour‐bearing GPx4 Tg mice when compared to wildtype mice*. Gastrocnemius and quadricep muscle masses normalized to body weight‐tumour were 20% lower in wildtype LLC‐tumour‐bearing mice compared to wildtype sham (Figure 3a,b). Gastrocnemius and quadricep muscle masses were higher in GPx4 Tg LLC‐tumour‐bearing mice when compared to wildtype LLC‐tumour‐bearing mice (Figure 3a,b). There was a main effect (ME) of genotype for higher TA masses in GPx4 Tg mice compared to wildtype mice (Figure 3c). Muscle wet weights that are not normalized to bodyweight‐tumour are shown in Figure S1. Figure 3d shows representative images of myosin heavy chain staining for cross‐sectional area analysis. The percent small (0–1000 μm^2^) type IIA fibres was higher in TA muscle from wildtype LLC when compared to GPx4 Tg LLC (Figure 3e). The amount of small type IIx fibres (0–1600 μm^2^) was higher in GPx4 Tg mice when compared to wildtype mice (Figure 3e).

### 3.2 Extensor Digitorum Longus Force Generation Is Impaired in LLC‐Tumour‐Bearing Mice

**FIGURE 4:**
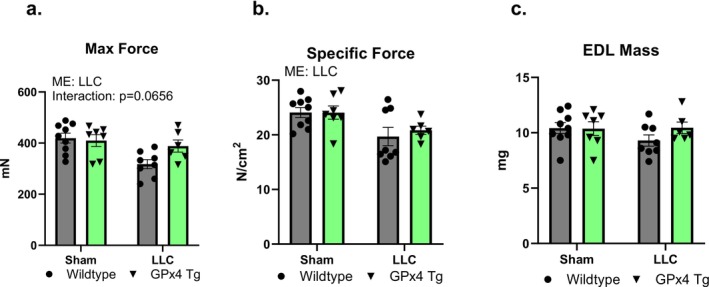
Extensor digitorum longus contractility in control and tumour‐bearing wildtype and GPx4 Tg mice. (a) Maximum force in EDL muscle from control and LLC‐tumour‐bearing wildtype and GPx4 Tg mice. (b) Specific force in EDL muscle from control and LLC‐tumour‐bearing wildtype and GPx4 Tg mice. (c) EDL masses for contractile experiment in control and LLC‐tumour‐bearing wildtype and GPx4 Tg mice. An *n* = 6–8 per group was used. Asterisk denotes post hoc differences at an alpha set at *p* < 0.05. ME: main effect.

We used EDL for muscle‐stimulated force generation ex vivo to assess muscle function in our experimental model. There was a main effect for LLC‐tumour‐bearing mice to have lower maximal and specific force generation (Figure 4a,b). EDL mass was not different among groups (Figure 4c).

### 3.3 GPx4 Overexpression Partially Improves Mitochondrial Respiration in LLC‐Tumour‐Bearing Mice Compared to Wildtype Mice

**FIGURE 5:**
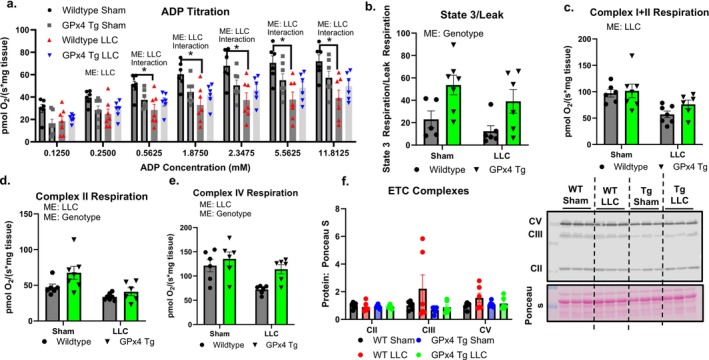
Mitochondrial respiration and proteins in control and tumour‐bearing wildtype and GPx4 Tg mice. (a) ADP titration measuring Complex I respiration in red gastrocnemius muscle from control and LLC‐tumour‐bearing wildtype and GPx4 Tg mice. (b) State 3 over leak respiration in red gastrocnemius muscle from control and LLC‐tumour‐bearing wildtype and GPx4 Tg mice. (c) Complex II respiration in red gastrocnemius muscle from control and LLC‐tumour‐bearing wildtype and GPx4 Tg mice. (d) Complex I + II respiration in red gastrocnemius muscle from control and LLC‐tumour‐bearing wildtype and GPx4 Tg mice. (e) Complex IV respiration in red gastrocnemius muscle from control and LLC‐tumour‐bearing wildtype and GPx4 Tg mice. (f) ETC complexes in gastrocnemius muscle from control and LLC‐tumour‐bearing wildtype and GPx4 Tg mice. (g) Representative images of western blots. An *n* = 5–8 per group was used. Asterisk denotes post hoc differences at an alpha set at *p* < 0.05. ME: main effect.

Complex I‐mediated respiration in permeabilized gastrocnemius fibre bundles was lower in wildtype LLC‐tumour‐bearing mice when compared to wildtype sham at concentrations of ADP ranging from 0.56–11.8 mM (Figure 5a). State 3 over leak respiration, an index of ATP synthesis efficiency, was higher in permeabilized gastrocnemius fibre bundles from GPx4 Tg mice than those from wildtype mice (Figure 5b). There was a main effect for respiration supported by both Complexes I + II in permeabilized gastrocnemius fibre bundles to be lower in LLC‐tumour‐bearing mice when compared to nontumour‐bearing mice (Figure 5c). There was a main effect for Complex II and Complex IV respiration to be lower in LLC‐tumour‐bearing mice compared to nontumour‐bearing controls (Figure 5d,e). There was a main effect for Complex II and Complex IV respiration to be higher in GPx4 Tg mice compared to wildtype mice (Figure 5d,e). However, despite these functional changes, the protein content of mitochondrial electron transport chain complexes was not different between groups (Figure 5f,g).

### 3.4 Muscle Oxylipin Profile Is Altered in LLC‐Tumour‐Bearing Mice

**FIGURE 6:**
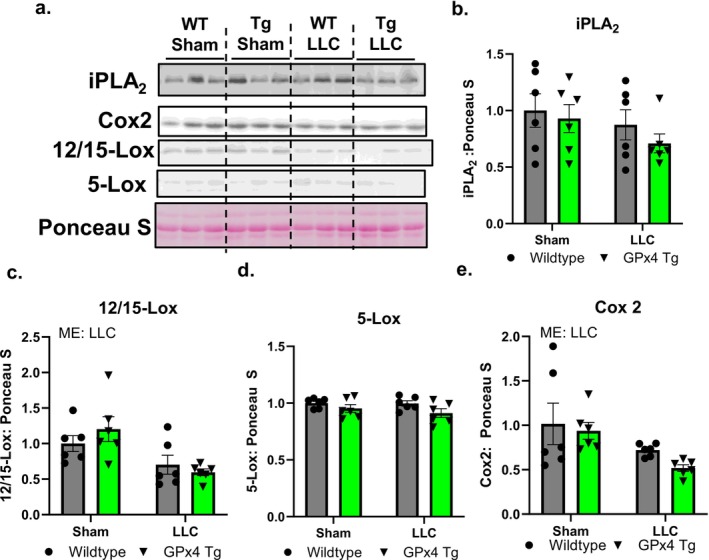
Enzymes that generate oxylipins in control and tumour‐bearing wildtype and GPx4 Tg mice. (a) Representative images of western blots. (b) iPLA_2_ protein content in gastrocnemius muscle from control and LLC‐tumour‐bearing wildtype and GPx4 Tg mice. (c) 12/15‐Lox protein content in gastrocnemius muscle from control and LLC‐tumour‐bearing wildtype and GPx4 Tg mice. (d) 5‐Lox protein content in gastrocnemius muscle from control and LLC‐tumour‐bearing wildtype and GPx4 Tg mice. (e) Cox‐2 protein content in gastrocnemius muscle from control and LLC‐tumour‐bearing wildtype and GPx4 Tg mice. An *n* = 6 per group was used. Asterisk denotes post hoc differences at an alpha set at *p* < 0.05. ME: main effect.

To better understand the potential role of oxylipins in skeletal muscle in response to cancer, we measured protein content of enzymes involved in oxylipin generation and lipid omics in gastrocnemius muscle. iPLA_2_ protein content was not different between groups (Figure 6a,b). Protein content for 12/15‐Lox was lower in gastrocnemius muscle from LLC‐Tumour‐bearing mice when compared to sham GPx4 Tg mice (Figure 6a,c). Gastrocnemius protein content for 5‐Lox was not different between groups (Figure 6a,d). Cox‐2 protein content was lower in LLC‐tumour‐bearing mice compared to sham (Figure 6a,e).

**FIGURE 7:**
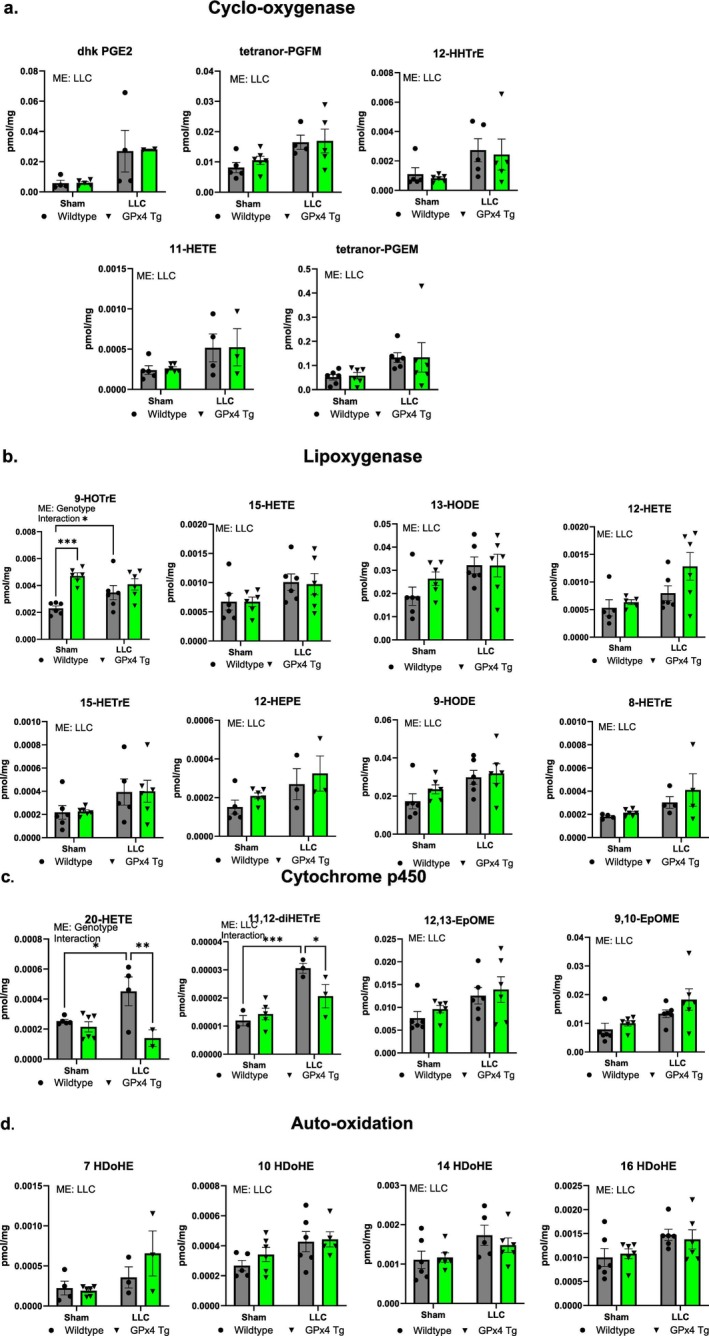
Oxylipins in control and tumour‐bearing wildtype and GPx4 Tg mice. (a) Oxylipins generated from cyclo‐oxygenase from gastrocnemius muscle from control and LLC‐tumour‐bearing wildtype and GPx4 Tg mice. (b) Oxylipins generated from lipoxygenase from gastrocnemius muscle from control and LLC‐tumour‐bearing wildtype and GPx4 Tg mice. (c) Oxylipins generated from cytochrome p450 from gastrocnemius muscle from control and LLC‐tumour‐bearing wildtype and GPx4 Tg mice. (d) Oxylipins generated from auto‐oxidation from gastrocnemius muscle from control and LLC‐tumour‐bearing wildtype and GPx4 Tg mice. An *n* = 6 per group was used. Asterisk denotes post hoc differences at an alpha set at *p* < 0.05. ME: main effect.

There was a main effect for dhk PGE2, tetranor‐PGFM, 12‐HHTrE, 11‐HETE and tetranor PGEM to be higher in gastrocnemius muscle from LLC‐tumour‐bearing mice when compared to sham (Figure 7a). 9‐HOTrE was significantly higher in gastrocnemius muscle from sham GPx4 Tg mice when compared to wildtype sham and wildtype LLC‐tumour‐bearing mice (Figure 7b). There was a main effect for 9‐HOTrE to be higher in GPx4 Tg mice when compared to wildtype mice (Figure 7b). There was a main effect for 15‐HETE, 12‐HETE, 13‐HODE, 15‐HETrE, 12‐HEPE, 9‐HODE and 8‐HETrE to be higher in gastrocnemius muscle from tumour‐bearing mice when compared to sham (Figure 7b). 20‐HETE was 2‐fold higher in gastrocnemius from wildtype LLC‐tumour‐bearing mice compared to wildtype sham and GPx4 Tg LLC‐tumour‐bearing mice (Figure 7c). There was a main effect for 20‐HETE to be lower in GPx4 Tg mice compared to wildtype mice (Figure 7c). 11,12‐diHETrE was significantly higher in gastrocnemius muscle from wildtype LLC‐tumour‐bearing mice when compared to wildtype sham and GPx4 Tg LLC‐tumour‐bearing mice (Figure 7c). There was a main effect for 11,12‐diHETrE, 12,13‐EpOME and 9,10‐EpOME to be higher in gastrocnemius muscle from LLC‐tumour‐bearing mice when compared to sham (Figure 7c). There was a main effect for 7 HDoHE, 10 HDoHE, 14 HDoHE and 16 HDoHE to be higher in gastrocnemius muscle from LLC‐tumour‐bearing mice when compared to sham (Figure 7d). All other oxylipins measured are shown in Figure S3.

### 3.5 Gene Expression Analysis in Quadricep Muscle

**FIGURE 8:**
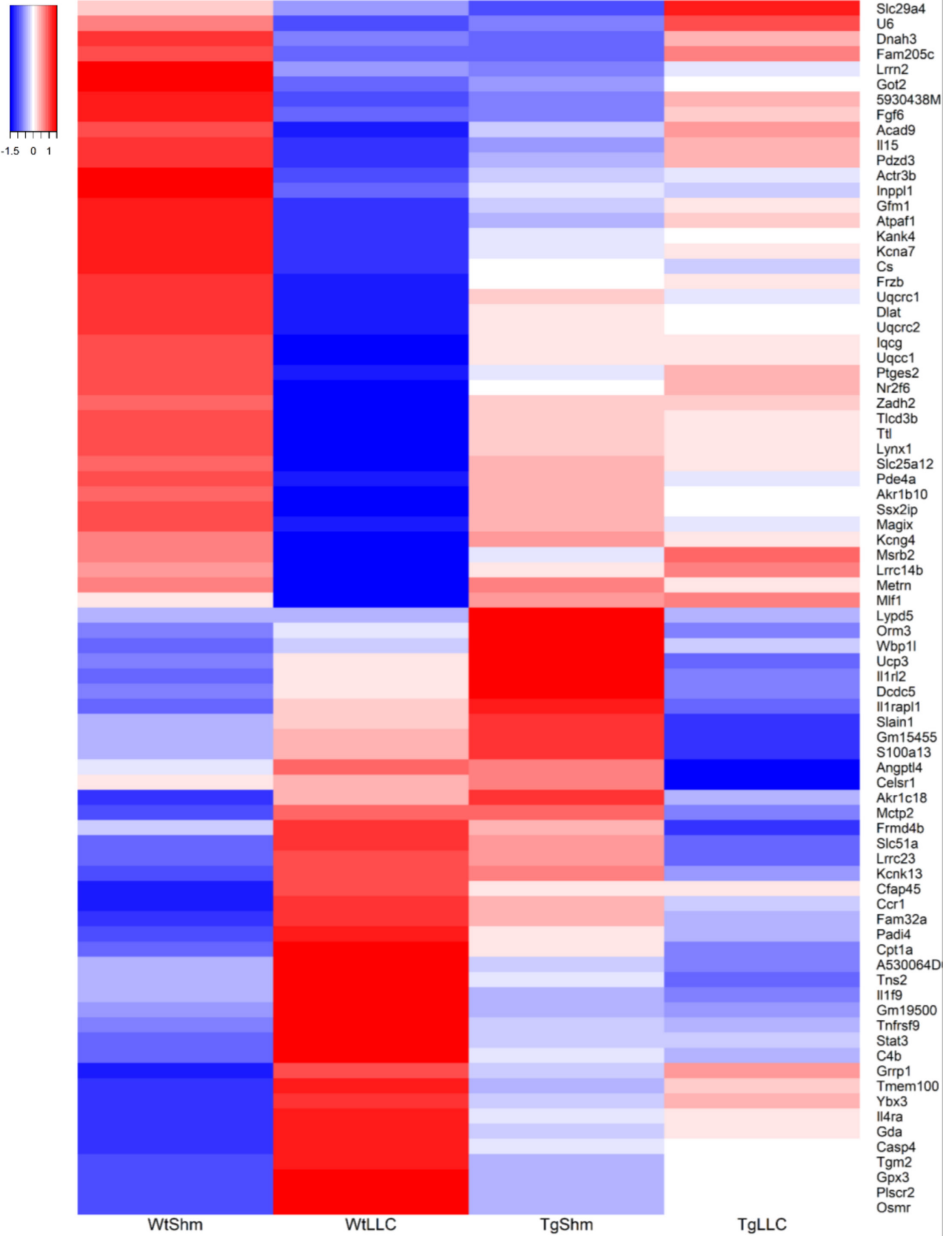
Transcriptomics in control and tumour‐bearing wildtype and GPx4 Tg mice. Top 80 genes that expression in quadricep muscle that was reversed when comparing WT LLC and Tg LLC. These genes were the top 80 lowest *p*‐values when detecting an interaction. An *n* = 6 per group was used.

To further explore the changes in muscle which may contribute to the protective effect of GPx4 in muscle from LLC‐tumour‐bearing mice, we performed transcriptomic analysis in quadricep muscle. Figure 8 shows the top LLC cancer–affected genes appear protected by GPx4 transgene overexpression. In wildtype mice, the strong colour contrast between the first two columns of the heat map reflects marked differential expression between cancer (WT LLC) and control (WT Sham) samples. In GPx4 Tg mice, this contrast between LLC and Sham samples is largely diminished (last two columns of the heat map). Notably, the expression profile of Tg cancer samples (Tg LLC, fourth column) more closely resembles that of healthy Wt Shm samples rather than Wt LLC cancer samples, highlighting the protective effect of GPx4 Tg overexpression; 112 genes differentially expressed between LLC and Sham samples, with strong modulation of the difference by the GPx4 Tg intervention (the interaction effect), were selected as matching this expression reversing profile. For clarity (gene symbol readability), the heat map is restricted to the 80 genes with the highest interaction effect.

**FIGURE 9:**
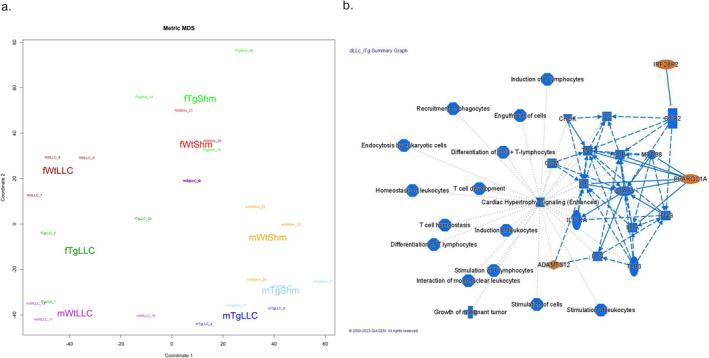
Transcriptomics in control and tumour‐bearing wildtype and GPx4 Tg mice. (a) PCA plot comparing gene expression in quadricep muscle form male and female control and tumour‐bearing wildtype and GPx4 Tg mice. (b) Pathway analysis showing genes associated with inflammation are lower in quadricep muscle when comparing WT LLC and Tg LLC. An *n* = 6 per group was used.

Figure 9a shows principal component representation (PCA) of gene expression profiles illustrating sample similarities and the protective effect of GPx4 transgene (Tg) expression against LLC‐induced transcriptional changes. Each point represents an individual sample. Labels are formatted compactly: the first letter indicates sex (f, female; m, male), the next two characters indicate genotype (Tg for GPx4 transgenic, Wt for wild type), and the final two characters indicate condition (Shm, healthy; LLC, cancer). Each sample is represented in small font label with the sample number as a suffix while large font labels show each mouse group centroid. Clustering reflects global expression similarities, with distinct separation between experimental groups highlighting differential transcriptomic profiles. First principal component strongly separate male mWtLLC (cancer) sample from mWtShm (healthy) samples, with both mTgLLC and mTgShm samples clustering nearby the healthy samples, consistent with strong LLC‐induced transcriptional reprogramming by GPx4 intervention. Some similar separation can be seen for female samples (fWtLLC versus the other three female phenotypes) along the second diagonal of the plot.

Figure 9B shows the graphical summary highlighting the key biological functions, canonical pathways, and upstream regulators inferred from the set of genes differentially expressed when comparing WT LLC and Tg LLC mice. The analysis was conducted using Ingenuity Pathway Analysis [25]. Orange shapes indicate predicted activation, while blue shapes denote predicted inhibition, based on IPA *z*‐scores, with colour intensity potentially indicating the degree of regulation in the datasets. Grey elements indicate entities for which no prediction could be made. Solid lines represent direct relationships; dashed lines indicate indirect interactions. The algorithm uses machine learning techniques to prioritize and connect entities, inferring relationships derived from the in‐house QIAGEN Knowledge Graph database.

### 3.6 GPx4 Overexpression Lowers Muscle Inflammation in LLC‐Tumour‐Bearing Mice When Compared to Tumour‐Bearing Controls

**FIGURE 10:**
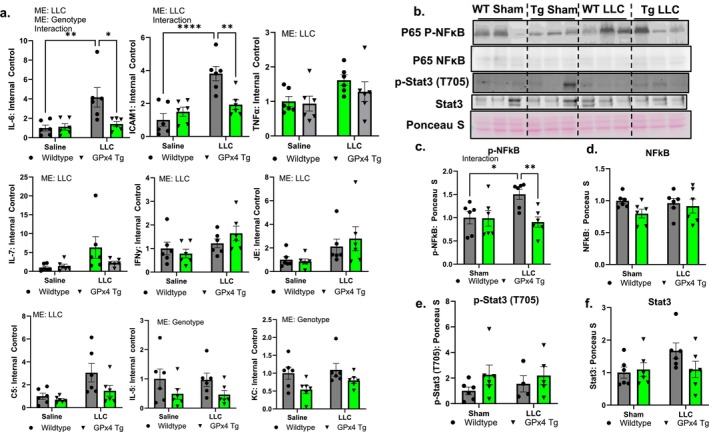
Protein level of cytokines, STAT3 and NFkB in quadricep from control and tumour‐bearing wildtype and GPx4 Tg. (a) Protein level of cytokines from quadricep muscle from control and LLC‐tumour‐bearing wildtype and GPx4 Tg mice. (a) IL‐6 protein content from cytokine array. (b) Representative western blot images. (c) Phospho p65 NFkB from quadricep muscle from control and LLC‐tumour‐bearing wildtype and GPx4 Tg mice. (d) p65 NFkB from quadricep muscle from control and LLC‐tumour‐bearing wildtype and GPx4 Tg mice. (e) Phospho STAT3 from quadricep muscle from control and LLC‐tumour‐bearing wildtype and GPx4 Tg mice. (f) STAT3 from quadricep muscle from control and LLC‐tumour‐bearing wildtype and GPx4 Tg mice. An *n* = 6 per group was used. Asterisk denotes post hoc differences at an alpha set at *p* < 0.05. ME: main effect.

Due to the changes in inflammatory pathways identified through our transcriptomic analysis, we further probed differences in inflammatory cytokines at the protein level. Protein content of the cytokines IL‐7, IL‐6, ICAM1, C5/C5a, JE, TNFa and IFN*γ* was higher in quadricep muscle from tumour‐bearing mice when compared to controls (Figure 10a). IL‐6, KC and IL‐5 were lower in quadricep muscle from GPx4 Tg mice when compared to wildtype mice (Figure 10a). There was a statistical interaction between groups in quadricep muscle for IL‐6 and ICAM1 (Figure 10a). IL‐6 and ICAM1 were ~4‐fold higher in wildtype LLC‐tumour‐bearing mice when compared to wildtype nontumour‐bearing mice (Figure 10a). Protein content of IL‐6 and ICAM1 was 2‐fold lower in GPx4 Tg LLC‐tumour‐bearing mice when compared to wildtype tumour‐bearing mice (Figure 10a). A heat map showing protein content of all cytokines measured is shown in Figure S4. Representative immunoblot images are shown in Figure 10b. p‐NFκB protein content was higher in gastrocnemius muscle from wildtype LLC‐tumour‐bearing mice when compared to wildtype controls while there was no change in total NFκB protein (Figure 10c,d). p‐NFκB protein content was lower in gastrocnemius muscle from GPx4 Tg LLC‐tumour‐bearing mice when compared to wildtype LLC‐tumour‐bearing mice with no difference in total NFκB protein (Figure 10c,d). There were no differences in gastrocnemius muscle p‐Stat3 and total Stat3 content between any of the groups (Figure 10e,f).

## 4 Discussion

In our study, GPx4 overexpression also mitigated cancer cachexia by protecting muscle mass. We also found that GPx4 overexpression lowered markers of lipid peroxidation in muscle when compared to wildtype mice. We found that Complex I mitochondrial respiration was lower in tumour‐bearing wildtype mice but not lower in tumour‐bearing mice with GPx4 overexpression. Oxylipins generated by 12/15‐Lox, 5‐Lox, Cyclo‐Oxygenase and Cytochrome P450 were higher in muscle and tumour‐bearing mice compared to nontumour‐bearing controls. Finally, we found that GPx4 overexpression lowered muscle inflammation transcriptome and IL‐6 protein content in tumour‐bearing mice compared to wildtype tumour‐bearing mice.

The introduction of carbonyl groups into the amino acid side chains of proteins is a major hallmark for oxidative damage and lipid peroxidation [26]. F_2_‐isoprostanes are produced because of the lipid peroxidation of arachidonic acid [27]. We found that GPx4 overexpression effectively lowered markers of lipid peroxidation (protein carbonyls and F_2_‐isoprostanes) in muscle when compared to wildtype mice.

Our experimental model showed a moderate cancer cachexia phenotype. We showed that overexpression of GPx4 mitigated LLC‐induced muscle atrophy in gastrocnemius, quadricep and TA muscles. We did not observe bodyweight loss despite muscle atrophy and subcutaneous fat loss. A potential reason why we did not observe bodyweight loss is because mass was higher in other organs. For example, spleen mass was ~3.5 times higher in tumour‐bearing mice when compared to cancer‐free controls. Prior work showed that type 2 fibres and fibres that do not stain positive for oxidative marker, succinate dehydrogenase, are more susceptible to atrophy in LLC‐tumour‐bearing mice [4]. We stained the TA muscle for different isoforms of myosin heavy chain and found that GPx4 overexpression protected type 2A fibres in LLC‐tumour‐bearing mice. To our surprise, GPx4 overexpression did not rescue skeletal muscle contractility as we did not observe an interaction between groups in both maximal force and specific force. GPx4 overexpression can mitigate muscle weakness during aging and oxidative stress‐induced frailty by improving membrane excitability and calcium sensitivity [18, 20]. If we had a more severe phenotype in our experimental model (refractory cachexia), GPx4 overexpression may protect muscle contractility in tumour‐bearing mice [28].

Lower mitochondrial respiration and higher mitochondrial ROS generation are associated with cancer cachexia [4]. Like our prior work [4, 6], we showed that muscle from LLC‐tumour‐bearing mice had lower mitochondrial respiration than nontumour‐bearing controls. We have shown that GPx4 overexpression improved muscle mitochondrial respiration in models of muscle frailty and sarcopenia [18, 20, 29], and other studies showed that neutralizing lipid hydroperoxides helped maintain mitochondrial function [30]. It is possible the lower respiration observed in wildtype LLC‐tumour‐bearing mice when compared to wildtype controls was driven by lower mitochondrial content because we normalized respiration measurements to fibre bundle mass rather than mitochondrial content. We did not observe a change in the content of mitochondrial electron transport chain proteins in powdered muscle. It should be noted that it is possible to have a change in biogenesis of mitochondria without a change in content (increased mitochondrial turnover). Our data show that GPx4 overexpression improves muscle Complex I mitochondrial respiration in LLC‐tumour‐bearing mice.

We performed transcriptomic analysis to show what pathways are differentially expressed in GPx4 Tg tumour‐bearing mice compared to wildtype tumour‐bearing mice. Consistent with prior data, we showed that cancer induced a localized muscle inflammatory transcript response [31, 32]. Localized muscle inflammation can lead to wasting of skeletal muscle through protein catabolism [32, 33]. We found that overexpression of GPx4 lowered the transcript of many pathways associated with inflammation in muscle from LLC‐tumour‐bearing mice when compared to tumour‐bearing wildtype mice. We also measured several cytokines in skeletal muscle at the protein level. We found that GPx4 overexpression lowered the protein level of many cytokines in muscle when compared to wildtype mice. Of particular importance, IL‐6 is a key mediator of cancer cachexia. IL‐6 can lead to muscle atrophy [34], dysfunction [35], and mitochondrial pathologies [36] in tumour‐bearing mice. Our data showed that IL‐6 protein was lower in muscle from GPx4 Tg tumour‐bearing mice when compared to muscle from tumour‐bearing wildtype mice. Lowering levels of muscle IL‐6 may serve as a protective mechanism in GPx4 Tg LLC‐tumour‐bearing mice. IL‐6 is predominantly regulated by STAT3 or NF κB in skeletal muscle. We showed that phosphorylation of NF κB was lower GPx4 Tg tumour‐bearing mice when compared to wildtype tumour‐bearing mice, while STAT3 phosphorylation and protein content were not different between groups. NF κB is a protein activated because of inflammation that is a key mediator of cancer cachexia [32]. NF κB activation can also contribute to localized muscle inflammation, so lowering NF κB phosphorylation may contribute to the GPx4‐mediated decrease in muscle inflammation.

We showed that gastrocnemius muscle from tumour‐bearing mice has an altered oxylipin profile when compared to nontumour‐bearing controls. Oxylipins are oxygenated products of polyunsaturated fatty acids that play a role in inflammatory responses among other regulatory roles [37]. Of note, oxylipins generated by 12/15‐Lox are higher in muscle from tumour‐bearing mice when compared to nontumour‐bearing controls. Our prior work shows that oxylipins generated by 12/15‐Lox may contribute to skeletal muscle pathologies [38]. The oxylipin 15‐HETE, a product of 12/15‐Lox, has been shown to trigger muscle loss via the ubiquitin proteasome system in vitro [39]. In contrast, our data show that 12/15‐Lox protein content is lower in LLC‐tumour‐bearing mice when compared to controls. Despite this, we observed changes in transcript and protein content in inflammatory pathways in our study. Other unknown regulatory mechanisms that alter the activity for enzymatic generation of oxylipins or auto‐oxidation may be occurring. Prior work has shown that ROS generation is higher in muscle from tumour‐bearing mice when compared to controls [4]. Our study showed several oxylipins associated with auto‐oxidation are higher in muscle from LLC‐tumour‐bearing mice when compared to controls. Therefore, it is possible that auto‐oxidation contributes to the altered oxylipin profile observed in our study.

Prior work shows that GPx4 overexpression can lower muscle oxylipin content [18]. However, in our study GPx4 overexpression does not change the muscle oxylipin content in muscle from LLC‐tumour‐bearing mice despite GPx4 overexpression lowering markers of lipid peroxidation (F_2_ isoprostanes and carbonyls) in muscle when compared to wildtype mice. GPx4 is typically found within plasma membranes. Therefore, GPx4 overexpression may have lowered lipid peroxidation within the sarcolemma.

There are a few limitations to our current study. We need to use more preclinical models of cancer cachexia. Cancer cachexia preclinical models have slightly different phenotypes and sometimes underlying mechanisms that contribute to cancer cachexia can vary from model to model. Cancer diagnosis occurs most commonly in aged individuals. Our preclinical model uses 6‐month‐old mice, which would be equivalent to a young adult in a patient population. Future studies will explore GPx4 overexpression in different cancer cachexia preclinical cachexia models and in age‐appropriate rodent models. We did not account for mitochondrial content in our respiration measurements, so changes observed in respiration may be a result of changes in mitochondrial content.

In summary, our study shows that GPx4 overexpression may mitigate cancer cachexia in LLC‐tumour‐bearing mice. GPx4 overexpression protected muscle mass and Complex 1 mitochondrial respiration, while lowering inflammatory transcript and IL‐6 protein in tumour‐bearing mice. These data suggest that targeting lipid hydroperoxides could be an effective strategy to mitigate cancer cachexia.

## Ethics Statement

The authors certify that they comply with the ethical guidelines for authorship and publishing of the Journal of Cachexia, Sarcopenia and Muscle [40].

## Conflicts of Interest

The authors declare no conflicts of interest.

## Supporting information


**Figure S1:** jcsm70255‐sup‐0001‐Supplementary_Figure.pptx. **Muscle wet weights. (a)** Gastrocnemius mass in control and LLC‐tumour‐bearing wildtype and GPx4 Tg mice. (b) Quadricep mass in control and LLC‐tumour‐bearing wildtype and GPx4 Tg mice. **(c)** TA mass in control and LLC‐tumour‐bearing wildtype and GPx4 Tg mice. (d) Soleus mass in control and LLC‐tumour‐bearing wildtype and GPx4 Tg mice. (e) EDL mass in control and LLC‐tumour‐bearing wildtype and GPx4 Tg mice. (f) Type I myofibers size comparison. An *n* = 8–11 per group was used. Asterisk denotes post hoc differences at an alpha set at *p* < 0.05. ME: Main Effect.
**Figure S2:** Organ wet weights. (a) Spleen mass in control and LLC‐tumour‐bearing wildtype and GPx4 Tg mice. (b) Subcutaneous fat mass in control and LLC‐tumour‐bearing wildtype and GPx4 Tg mice. (c) Epidydimal fat mass in control and LLC‐tumour‐bearing wildtype and GPx4 Tg mice. (d) Brain mass in control and LLC‐tumour‐bearing wildtype and GPx4 Tg mice. (e) Heart mass in control and LLC‐tumour‐bearing wildtype and GPx4 Tg mice. (f) Liver mass in control and LLC‐tumour‐bearing wildtype and GPx4 Tg mice. An *n* = 8–11 per group was used. Asterisk denotes post hoc differences at an alpha set at *p* < 0.05. ME: Main Effect.
**Figure S3:** Oxylipins in control and tumour‐bearing wildtype and GPx4 Tg mice. (a) Oxylipins generated from lipoxygenase from control and LLC‐tumour‐bearing wildtype and GPx4 Tg mice. (b) Oxylipins generated via auto‐oxidation from control and LLC‐tumour‐bearing wildtype and GPx4 Tg mice. (c) Oxylipins generated from cytochrome p450 from control and LLC‐tumour‐bearing wildtype and GPx4 Tg mice. (d) Oxylipins generated from cyclo‐oxygenase from control and LLC‐tumour‐bearing wildtype and GPx4 Tg mice. An *n* = 6 per group was used. Asterisk denotes post hoc differences at an alpha set at *p* < 0.05. ME: Main Effect.
**Figure S4:** Heat map of cytokines measured. (a) Heat map showing all cytokines measured. An *n* = 6 per group was used.

**Data S1:** Supporting information.
