# Supplementary material for: Phospholipid Glutathione Peroxidase Overexpression Mitigates Cancer Cachexia by Protecting Muscle Mass and Lowering Inflammation
Source: J Cachexia Sarcopenia Muscle. 2026 Mar 19;17(2):e70255. doi: 10.1002/jcsm.70255 (PMC13140832; doi:10.1002/jcsm.70255)

## Slide 1
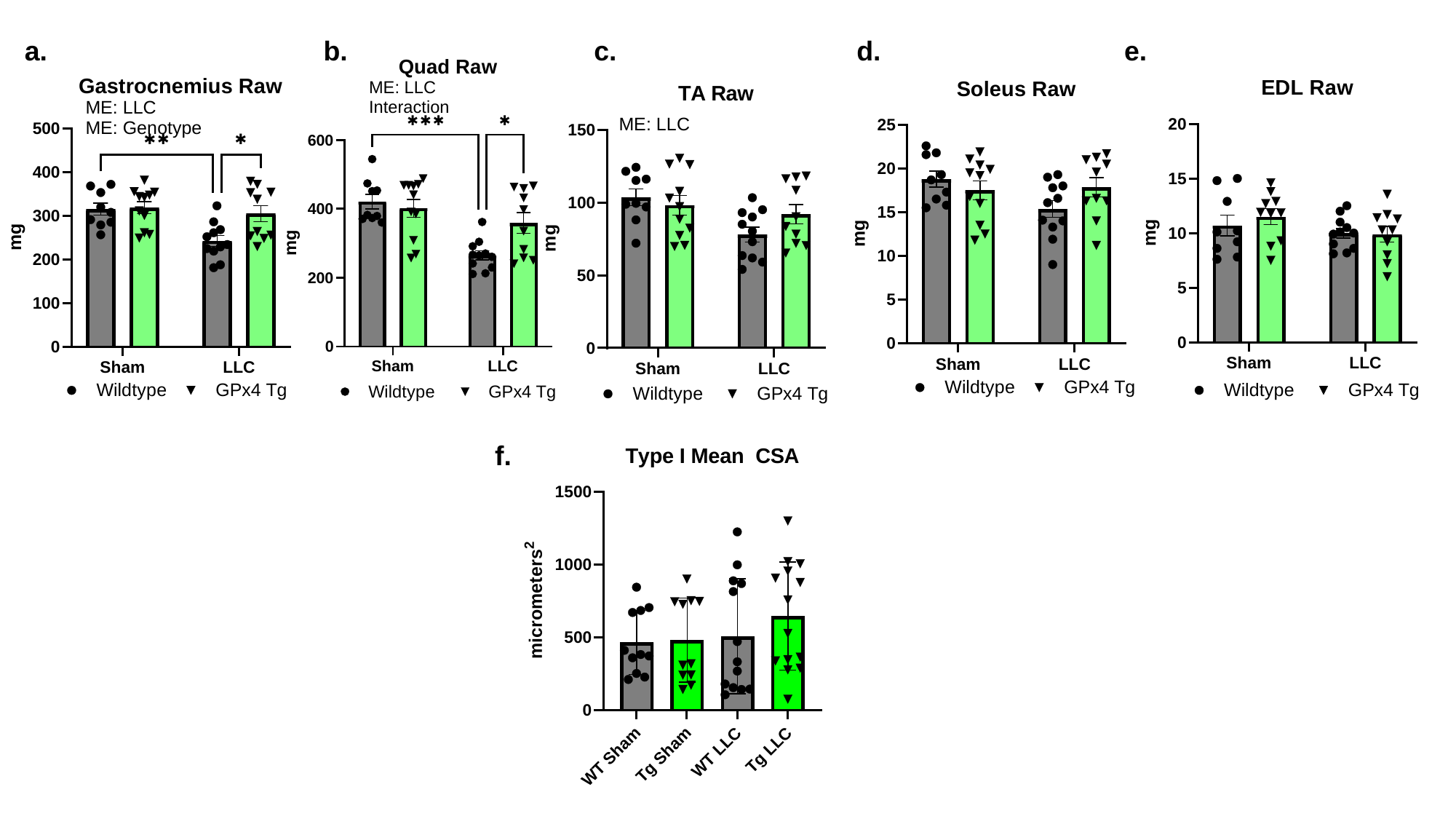

a.
b.
c.
d.
e.
f.

## Slide 2
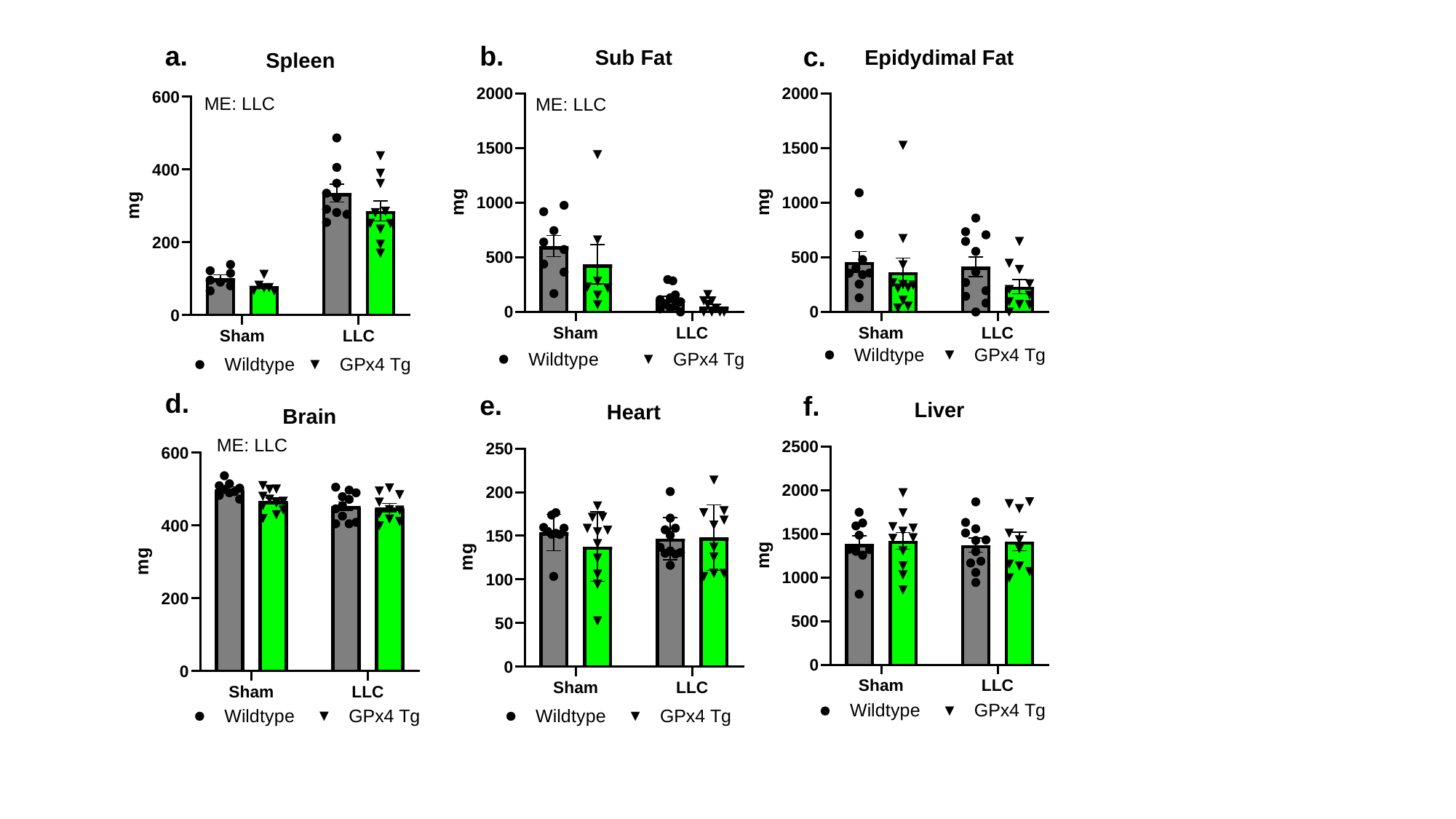

a.
b.
c.
d.
e.
f.

## Slide 3
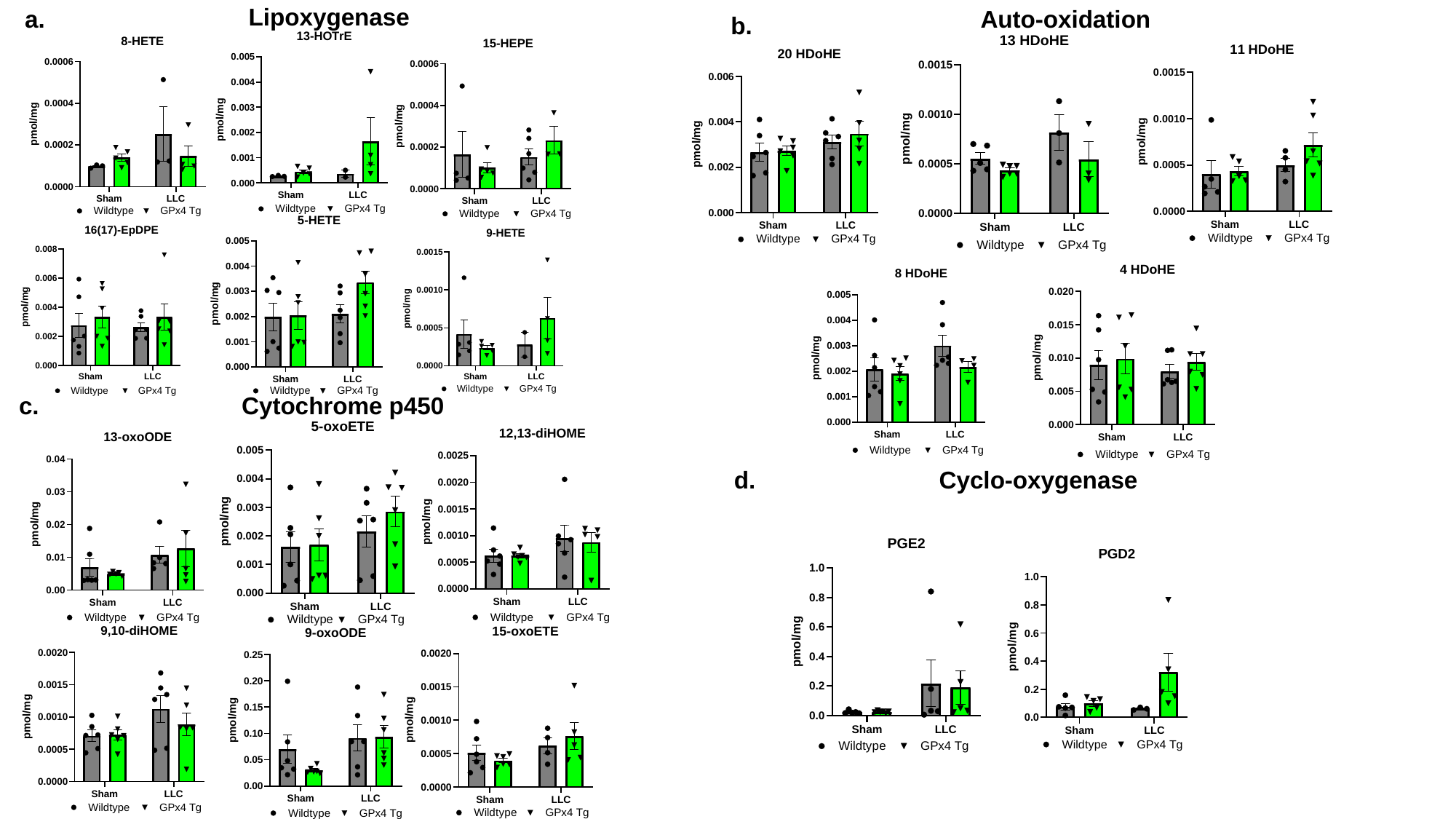

Lipoxygenase
a.
Auto-oxidation
b.
c.
Cytochrome p450
d.
Cyclo-oxygenase

## Slide 4
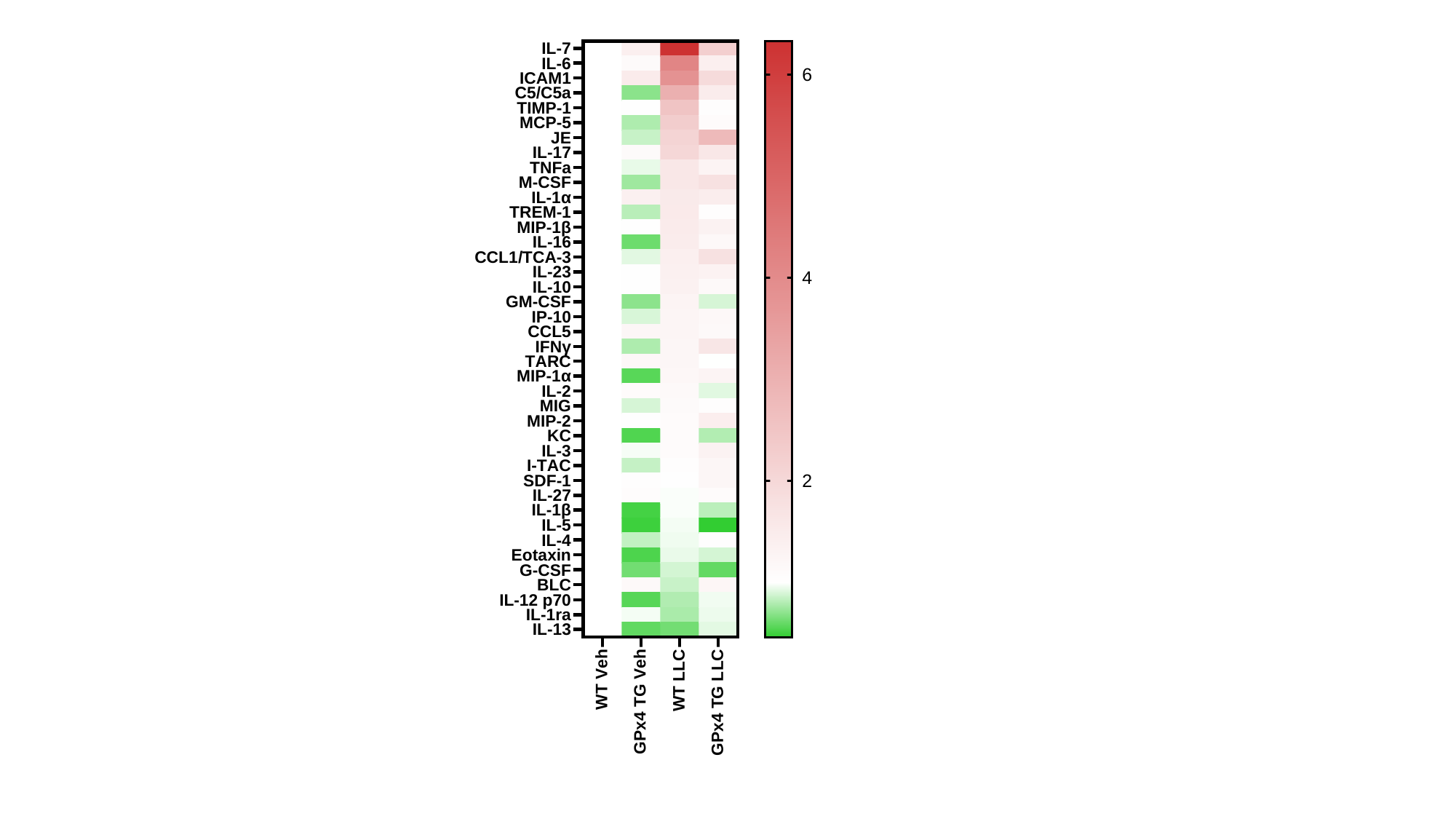

Supplement: Supplementary file 1 — Figure S1: jcsm70255‐sup‐0001‐Supplementary_Figure.pptx. Muscle wet weights. (a) Gastrocnemius mass in control and LLC‐tumour‐bearing wildtype and GPx4 Tg mice. (b) Quadricep mass in control and LLC‐tumour‐bearing wildtype and GPx4 Tg mice. (c) TA mass in control and LLC‐tumour‐bearing wildtype and GPx4 Tg mice. (d) Soleus mass in control and LLC‐tumour‐bearing wildtype and GPx4 Tg mice. (e) EDL mass in control and LLC‐tumour‐bearing wildtype and GPx4 Tg mice. (f) Type I myofibers size comparison. An n = 8–11 per group was used. Asterisk denotes post hoc differences at an alpha set at p < 0.05. ME: Main Effect. Figure S2: Organ wet weights. (a) Spleen mass in control and LLC‐tumour‐bearing wildtype and GPx4 Tg mice. (b) Subcutaneous fat mass in control and LLC‐tumour‐bearing wildtype and GPx4 Tg mice. (c) Epidydimal fat mass in control and LLC‐tumour‐bearing wildtype and GPx4 Tg mice. (d) Brain mass in control and LLC‐tumour‐bearing wildtype and GPx4 Tg mice. (e) Heart mass in control and LLC‐tumour‐bearing wildtype and GPx4 Tg mice. (f) Liver mass in control and LLC‐tumour‐bearing wildtype and GPx4 Tg mice. An n = 8–11 per group was used. Asterisk denotes post hoc differences at an alpha set at p < 0.05. ME: Main Effect. Figure S3: Oxylipins in control and tumour‐bearing wildtype and GPx4 Tg mice. (a) Oxylipins generated from lipoxygenase from control and LLC‐tumour‐bearing wildtype and GPx4 Tg mice. (b) Oxylipins generated via auto‐oxidation from control and LLC‐tumour‐bearing wildtype and GPx4 Tg mice. (c) Oxylipins generated from cytochrome p450 from control and LLC‐tumour‐bearing wildtype and GPx4 Tg mice. (d) Oxylipins generated from cyclo‐oxygenase from control and LLC‐tumour‐bearing wildtype and GPx4 Tg mice. An n = 6 per group was used. Asterisk denotes post hoc differences at an alpha set at p < 0.05. ME: Main Effect. Figure S4: Heat map of cytokines measured. (a) Heat map showing all cytokines measured. An n = 6 per group was used. [file JCSM-17-e70255-s002.pptx]
